# Assessment of areca nut use, practice and dependency among people in Guwahati, Assam: a cross-sectional study

**DOI:** 10.3332/ecancer.2021.1198

**Published:** 2021-03-04

**Authors:** Arvind Kumar, Kunal Oswal, Ravikant Singh, Neha Kharodia, Akash Pradhan, Lakshman Sethuraman, Ramachandran Venkataramanan, Arnie Purushotham

**Affiliations:** 1 Doctor for You (DFY), Mumbai 400043, Maharashtra, India; 2 Tata Trusts Cancer Care Program, Mumbai 400005, India; 3 King’s College London, Strand, London WC2R 2LS, United Kingdom

**Keywords:** areca nut, prevalence, practice, betel quid dependence scale (BQDS)

## Abstract

**Background:**

Areca nut is the fourth most commonly used psychoactive substance worldwide after tobacco, alcohol and caffeine. In India, it is perceived in various ways, ranging from a ‘fruit of divine origin’ in Hindu religious ceremonies to a mouth freshener. Areca nut use both on its own and with tobacco additives is addictive. The aim of this study was to understand the pattern of areca nut consumption, to determine the Knowledge, Attitude and Practices (KAP) among areca nut users and the dependency associated with areca nut use.

**Methods:**

A cross sectional study was conducted in Guwahati, Assam using a self-administered questionnaire eliciting the pattern of areca nut consumption, KAP among users and understanding their dependency using Betel Quid Dependence Scale. The chewers of areca nut alone with or without betel quid, gutkha and tobacco participated in the study. Areca nut users were selected using purposive sampling method from the vendor shops of all the four assembly areas of the city. Their participation was voluntary and free not to answer or quit the survey. The data was analysed using SPSS software.

**Results:**

A total of 500 participants were approached in all four areas, 479 completed the survey (response rate 95%). The people who participated in the study were mostly male with an average age of 40 years, educated to secondary level or higher, married and self-employed. Betel quid with tamul was the most prevalent form of areca nut chewing in both men and women. About 441 (92%) participants experienced pleasure when chewing areca nut and 327 (68%) chewed it to relieve stress. Only 86 (18%) of subjects had ever tried to quit chewing areca nut and 387 (81%) thought that it was highly addictive. The results revealed relatively high levels of endorsement for ‘physical and psychological urgent need’ (mean = 43%) and ‘increasing dose’ (mean = 50%), whereas endorsement level for ‘maladaptive use’ was low (mean = 16%).

**Conclusion:**

Areca nut use (tamul) is of major concern in India and many Southeast Asian countries and its use has been increasing across the globe. The evidence suggests a dependence similar to tobacco use and policy makers need to refine its strategy for control of its use by engaging with multiple stakeholders and adapting it to local context with surveillance and cessation guidelines in order to address this issue.

## Background

Areca nut is estimated to be used by 600 million people, particularly among South-east Asian communities [1–4]. Epidemiological surveys have estimated areca nut use in 20%–40% of the population above the age of 15 years in India, Nepal and Pakistan [5]. Areca nut, often referred to as the betel nut (as it is commonly chewed along with the Piper betel leaf), is the seed of the endosperm of the oriental palm [4, 6]. The areca nut and juice play an important ceremonial and cultural role in many countries including Myanmar, the Solomon Islands and Vietnam. It is common practice to offer these products to guests at important social gatherings, weddings and other religious events [3, 4]. Due to this cultural tradition, the use of the areca nut is widespread and considered a part of daily life, even among women and young children. In southern Asia, it is perceived to have medicinal values [4], including as an aphrodisiac, appetite suppressant, digestive aid and diuretic. Areca nut is often used in India as an alternative means of treatment in many diseases such as asthma, cough, dermatitis (topical), fainting, glaucoma, impotence, intestinal worms, leprosy, toothache, leucorrhoea (vaginal discharge) and vaginal laxity [4]. It is often used as self-medication for the alleviation of symptoms.

Areca nut can be chewed raw or processed by roasting, drying in the sun, soaking or boiling prior to chewing [6]. Other than betel nut, Areca has also been known as ‘kwai’ and ‘gue’ in Meghalaya. In Assam and Nagaland, it is known as ‘tamul’, whereas in Manipur and Mizoram it is ‘kua’ and ‘kuhva’ [6]. In India, different varieties of Areca nut preparations include *Neetadaka, Chali* or *kottapak, Parcha (Pareha), Kalipak, Iylon, Nayampak, Nuli, Supari, Tamol* and *Bura Tamul*. *Chikani* and *Bura tamul* are commonly used forms in Assam. *Bura Tamul* is prepared by preserving ripe fresh nuts for 3 to 4 months covered with bark from the betel tree, cow dung and soil which results in a moist chew [6, 7]. In the paper the terms Tamul and Areca nut have been used synonymously.

In most basic form, betel quid is a combination of betel leaf, areca nut and slaked lime (aqueous calcium hydroxide paste). Betel quid has a relaxing and stimulant effect by acting on the autonomic nervous system [1]. If tobacco is not added, areca nut is the main psychoactive substance in the betel quid [5].

Areca nut contains several alkaloids belonging to the pyridine group, the most important being arecoline. The others are arecaidine, guvacine and isoguvacine [4]. Nitrosated derivatives of arecal alkaloids are associated with increased risk of cancer [8]. Betel quid has been classified as a Group 1 carcinogen by the International Agency for Research on Cancer, while chewing areca is the single most important aetiologic factor for the development of oral submucous fibrosis. It has also shown to increase the risk of development of oral squamous cell carcinoma especially when the quid contains tobacco [4]. The malignant transformation of the Oral Submucous Fibrosis (OSF) was observed from 3% to 7.6 % [9, 10].

The stimulant and anxiolytic effects of areca have been associated with escalation of its use and dependency. However, disentangling the independent effects of areca addiction is challenging, given that many users concomitantly use tobacco [2]. Overall, areca nut plays multiple important roles in the socio-cultural context and economic conditions of the people in Assam. The aim of the study was to understand the pattern of areca nut consumption in Assam, India, to determine the knowledge, attitude and practice (KAP) among areca nut users and their dependency on areca nut.

## Methodology

A cross sectional study was conducted among areca nut users in the city of Guwahati (Assam), during March–April 2018. Guwahati (Dispur) is the capital of Assam and the largest city in North-East India. The city is divided into four legislative assemblies (Dispur, Jalukbari, Guwahati East and Guwahati West). Participants were included from all these four areas. A nonprobability purposive sampling method was planned to produce a sample that can be assumed to be representative of the population having a shared characteristic. The sample size was calculated based on the following assumption: 50% prevalence, 95% confidence level and 5% margin of error using the following formula *N*= 4pq/d^2^. The estimated sample size required was 384 subjects. The chewers of areca nut alone with or without betel quid, gutkha and tobacco were considered for this study.

Twenty-six vendor shops of all the four areas were approached and users who agreed to participate in the survey were included. All the participants were briefed about the aim, objectives and tools for around 2 minutes and mentioned that they are free to quit anytime. The correct understanding of the participants has been confirmed, and they encouraged to complete every item in order without skipping any. The principal investigator assisted with three interns from Tata Institute of Social Sciences, Guwahati collected the data. It took 20–25 minutes to complete one questionnaire.

A pre-tested close-ended questionnaire was used to elucidate the pattern of consumption. Special focus was on assessing three domains of learned behaviour towards tamul, viz. knowledge (cognitive), attitude (affective) and practice (psychomotor). The questionnaire was used to understand the prevalence, KAP pertaining to areca nut use and dependence assessment using Betel Quid Dependence Scale (BQDS). The validated BQDS, is a 16-item scale and employs a dichotomous outcome (Yes/No), used to assess the dependence on areca nut use. BQDS is a three-factor structure scale focussing on ‘physical and psychological urgent need’ (seven items), ‘increasing dose’ (five items) and ‘maladaptive use’ (four items) [9]. The BQDS scale was analysed by scores range from 0 to 1, so that each score represented the proportion of items endorsed (e.g. a score of 0.50 would mean that half of items were endorsed). The questionnaire was translated into Assamese and Hindi. Informed consent was sought from the eligible participants before data collection in their preferred language, either Assamese or Hindi. Confidentiality (no identifiers) and privacy (auditory) were maintained during and after the study.

### Statistical analysis

Survey data analysis involved descriptive statistics of the participants with demographics, areca nut use, knowledge and attitude and BQDS score. Point estimates were calculated with 95% confidence intervals for all measures (percentages). SPSS version 15 was used to analyse the data.

### BQDS score analysis

The total score of the BQDS ranged from 0 to 16 and the median (interquartile range) score was calculated in the study population [11]. The score ≥ 4 indicated dependence on areca nut. The percentage of participants who endorsed each item in the current study is presented in addition to rank order of each item (the most frequently endorsed item is ranked as ‘1’).

## Results

The questionnaire was administered to 500 participants, out of which 479 agreed to participate generating a response rate of 96%. The results of the study revealed that areca nut was consumed in four different formulations, namely, tamul/areca alone, betel quid (tamul with betel leaf), betel quid with tobacco and gutkha (gutkha = pan masala (nut) + tobacco). The study participants’ age ranged between 18 and 80 years (mean = 40 years), and age at initiation of areca habit ranged from 12 to 20 years (mean = 15 years). The duration of use of the product ranged from 10 to 30 years (mean = 18 years) and the frequency of areca nut use ranged from 3 to 6 times per day (mean = 4) (Table 1). On an average 100 Rs ($1.4) were spent daily with a range from 70 to 200 Rs/day ($1–$2.8) (Table 1). Betel quid with tamul was the most prevalent form of areca chewing in both men and women followed by tamul alone. Gutkha with tamul was found to be the least common habit among all age groups. Among the four different formulations of areca as mentioned above, betel quid with tamul was the most common variant chewed among all the occupational groups (Table 2).

### Knowledge pertaining to areca use among the study participants

Tamul was perceived to have beneficial effects by 108 (23%) participants, while 208 (43%) perceived tamul to have harmful effects on health (Table 3). Most of the participants felt that tamul did not cause staining of teeth and agreed that tamul was addictive. Approximately, a quarter of participants had noticed sores, white patches or gum problems at the site of tamul placement. Participants believed that tamul can cause oral cancer and almost half of them were aware that oral cancer is preventable. In all, less than half of the total participants were aware about a government programme for prevention of cancer.

### Attitude pertaining to areca nut use among the study participants

Majority of the participants experienced pleasure when chewing areca nut and chewed it to relieve stress (Table 3). Only 18% of participants had ever tried to quit chewing areca nut, while 78% were prepared to quit if they were made aware of harmful effects of areca nut use.

### Practices pertaining to areca nut use among the study participants

In all, majority of users indicated that their family members used areca nut (Table 3). Only a few users said they were thinking of quitting use of areca nut or had tried to quit in the last 6 months and 77% of participants indicate they would quit if they were informed that areca nut causes cancer. Majority of participants were willing to undergo oral cancer screening.

### BQDS analysis

Among the study population, 10% reported a total score of 0 points (without any dependent symptoms), 20% scored 1–3 points and 70% scored 4 points or above (Table 4). The results reveal relatively high levels of endorsement for ‘physical and psychological urgent need’ (mean percentage = 43%) and ‘increasing dose’ (mean percentage = 50%), whereas endorsement level for ‘maladaptive use’ was low (mean percentage = 16%). Most participants reported of having strong craving and felt they could not do without tamul.

## Discussion

The study highlights the use of areca nut in the study participants, pattern of use and its perceived effect and dependence on overall health. The study revealed areca nut was consumed in four different variations. There was reported an early age of initiation among the study participants with an increased frequency and duration of use. Most of the participants agreed that they derive pleasure and use areca nut to relieve stress. They also found it to be highly addictive and difficult to quit. The influence of family members using areca nut influenced use, but users showed a willingness to quit if services were available. The BQDS showed high levels of endorsement for ‘physical and psychological urgent need’ and increasing dose whereas endorsement level for maladaptive use was low.

The current study revealed that overall, tamul with betel quid was the most commonly consumed form of practice which is similar to findings reported by Sein *et al* [12], that there were different patterns of chewing areca and the most common form was chewing betel quid, which usually consists of a leaf of betel-vine, areca nut, slaked lime and some aroma [10]**.**

In the present study, chewing areca nut in any form was almost 2.3 times more prevalent in men than women. Most of the studies report this difference to be lower while some studies found that the use of areca nut was more common in women. In a study conducted on 99,598 permanent residents of Mumbai, who were 35 years or older [5], areca nut in all forms was used by 29.7% of women and 37.8% of men [14], while in a study conducted in Karachi, Pakistan [13], 27.9% of men and 37.8% of women chewed areca nut in the form of betel quid.

In the present study, the average age of initiation to chew tamul was 15 years with a mean duration of use being 18 years. In a survey conducted by Schonland and Bradshaw [15], amongst Natal Indians with special reference to betel quid chewing habits, the age at which chewing initiated was between 20 and 24 years. Two-fifths of chewers began the habit before the age of 20 years and a negligible number after the age of 40 years [15].

Self-employed groups contributed to 32% of study population while only 3% of them were homemakers. In this study, we observed a significant difference in areca use by occupation and ethnicity, whereas a study conducted among 589 Taiwanese prison inmates on betel nut chewing among males showed no correlation between occupation, ethnicity and betel nut chewing behaviour [16].

The knowledge questionnaire revealed that more than half of the study population were aware that tamul can have harmful effects on health and almost 80% believed it to have addictive properties. Tamul was perceived to have beneficial health effects by 22.5% of participants. This result is in contrast to a study conducted in Dakshina Kannada district of Karnataka on 90 areca nut chewers, wherein, the majority of respondents (69%) thought that chewing had beneficial effects and only a third of the sample knew about harmful effects of chewing [17]. In a further study in 2005 which surveyed 59 daily areca-only chewers from Karnataka State, India, areca nut chewing appeared to be perceived as beneficial when consumed in moderate amounts and having addictive properties equal to caffeine [18]. Chewing was largely viewed as a healthy practice if tobacco was not introduced into the quid.

Approximately one fourth of the participants had noticed sores, white patches or gum problems at the site of tamul placement at some point of time. There is enough literature evidence to demonstrate that betel nut has deleterious effects on oral soft tissues [19]. These deleterious effects include benign to precancerous and malignant changes in the oral mucosa. A review of betel quid chewing in mainland China reported the prevalence of oral sub mucous fibrosis among betel quid chewers ranged from 0.9% to 4.7% [20]. More than 60% participants believed that tamul could cause oral cancer while almost 50% of them felt oral cancer is preventable. There is sufficient evidence in humans for the carcinogenicity of betel quid with or without tobacco [14]. A hospital based case control study conducted by Mahapatra *et al* [21], showed that out of 134 cases of oral cancer ad 268 controls, adjusted odds ratio for getting oral cancer among supari users was 11.4, 6.4 for betel quid, 6.0 for chewing tobacco and 5.1 for gutka users. Dikshit and Kanhere [22] conducted a case control study in Bhopal and showed that out of 32 (13%) cases and 152 (8%) of control, odds of oral cancer was 1.7.

In the current study, more than 90% of participants used areca nut for pleasure and 70% believed it relieved their stress. In a study conducted among the population in North India that included 1,500 young college students aged between 15 and 22 years, the most common reason put forth by users of areca nut was peer pressure, followed by advertisements, general stress and academic pressure [23]. The reported effects of areca nut chewing included relaxation, improved concentration, mild lifting of mood and enhanced satisfaction after eating [10].

Almost 82% of users revealed areca nut chewing was practised by their family members. In a cross-sectional school-based survey of 2,200 adolescents from 26 schools in Karachi, Pakistan, participants considered it to be rude not to chew if their friends or family members were chewing betel quid [3]. This supports the fact that areca nut chewing is often influenced by someone in the family or in peer group.

Despite the awareness that areca nut can cause oral cancer, the attempted quit rate among participants was relatively low. This may be an indication, well supported by the literature, that users find it difficult to quit these behaviours [5]^,^ as betel quid shares many features with smokeless tobacco and both products are addictive [4]. The BQDS is the first instrument designed specifically to measure betel quid dependence [11]. In the present study, the results reveal relatively high levels of endorsement for ‘physical and psychological urgent need’ and ‘increasing dose’ whereas endorsement level for ‘maladaptive use’ was low.

## Conclusion

In conclusion, the current study demonstrates that preventive efforts need to focus upon the attitude of users alongside increasing their knowledge on the harmful effects of areca use. Areca nut is the fourth most commonly used psychoactive substance and chewing is a socially acceptable and widely practised habit amongst youth. Participants were not aware about the harmful effects of areca nut use. Long term use has potential to change from precancerous condition to malignancy.

### Recommendation

The behaviour change communication about tamul and effect on health can be communicated through the National Service Scheme, religious leaders, woman groups and community-based organisations. The dependence level is evident among the participants with difficulty in quitting and increasing its dosage to achieve desired effect. There is an urgent need to integrate areca nut use cessation services in the guidelines for cessation, develop policy measures on reducing the demand by communication and behaviour change strategies, and change the norms of its use in the community.

### Strength of the study

There are very few studies that describe areca nut user’s consumption pattern, knowledge, attitudes and dependence level among the general population. The current study included a broad range of users in terms of gender, age, education and occupation and explored pattern, KAP and dependence on the same population.

### Limitations of the study

The sample of chewers of areca nut was selected purposively so it may not be generalisable to the rest of India. Some of the respondents had difficulty recalling their ages and the exact year when they started chewing of areca nut, which may lead to recall bias. The cross-sectional design of the study precludes conclusions regarding causality of areca nut and dependence. Future longitudinal research is needed to assess the predictive validity of the BQDS.

### Study implication for policy

The study results have some implications on policy and practice of the use of tamul. Misconceptions regarding use of tamul need to be tackled through culturally sensitive messaging via multiple channels of communication. There is a need to develop a policy that may help to assist tamul chewers to quit as well as bring attention to the often-neglected issue of oral cancer, which is the commonest cancer among males and fourth most common in females in India. Furthermore, there is a need to encourage members of the community to get themselves screened for oral cancer under the national cancer screening programme.

## Funding

None.

## Conflicts of Interest

None.

## Declarations

None.

## Figures and Tables

**Table 1.** The distribution of the socio-demographic and chewing practice of the survey population in Guwahati, Assam (*N* = 479).

S.No.	Characteristics		Frequency *N* = 479 (%)
1	Age	18–30	142 (30)
		31–40	164 (34)
		41–50	96 (20)
		≥51	77 (16)
2	Gender	Male	341 (71)
		Female	138 (29)
3	Education	No formal education	59 (12)
		Primary	68 (14)
		Secondary	97 (20)
		Higher secondary	85 (18)
		Graduate and above	121 (26)
		Not recorded	49 (10)
4	Marital status	Married	341 (71)
		Single	138 (29)
5	Occupation	Self employed	152 (32)
		Daily wage	108 (23)
		Tamul seller	83 (18)
		Student	66 (14)
		Professional	54 (12)
		Homemakers	16 (04)
6	Age of initiation (in years)	15 (mean)	(12–20)
7	Duration of use (in years)	18 (mean)	(10–30)
8	Frequency of use (times per day)	4 (mean)	(3–6)
9	Money spent (per day in Rupees)	100 (mean) ($1.4)	(70–200) ($1–$2.8)

Mean age (SD) 38 (11.7), min–max, 18–80, 1$ = 70 INR

**Table 2.** Distribution of pattern of tamul consumption by product type and socio-demographic characteristics of the survey participants (*N* = 479).

Variable	Type of chewer (*N* = 479)									
	Tamul only		Betel quid with tamul		Betel quid with tamul and tobacco		Gutka		All	
	*n*	%	*n*	%	*n*	%	*n*	%	*n*	%
Age										
18–30	32	23	40	28	21	15	24	17	25	18
31–40	28	17	61	37	24	15	21	13	30	18
41–50	22	23	32	33	16	17	6	6	20	21
≥51	22	29	30	39	10	13	2	3	13	17
**Gender**										
Female	21	15	60	43	14	10	2	1	41	20
Male	83	24	103	30	57	17	51	15	47	14
**Marital status**										
Married	74	22	123	36	52	15	28	8	64	19
Single	30	22	40	29	19	14	25	18	24	17
**Occupation**										
Students	14	21	15	23	12	18	9	14	16	24
Home maker	2	13	11	69	3	18.8	0	0	0	0
Professional	11	20	20	37	7	13	1	2	15	28
Tamul seller	14	17	15	18	27	33	18	22	9	11
Self employed	38	25	53	35	15	9.9	17	11	29	19
Daily wage labourer	25	23	49	45	7	6.5	8	7	19	18

**Table 3.** Frequency and percentage of various responses to questions (*N* = 479).

Domain	Items	Yes *N* (%)	No *N* (%)
Knowledge	Do you perceive Tamul to have beneficial effects?	108 (23)	371 (77)
	Do you feel Tamul causes staining of teeth?	27 (6)	452 (94)
	Are you aware of harmful effects of Tamul?	208 (43)	271 (57)
	Do you think Tamul leads to addiction?	387 (81)	92 (19)
	Ever noticed abnormalities in mouth where Tamul is placed?	128 (27)	351 (73)
	Do you think Tamul causes oral cancer	305 (64)	174 (36)
	Do you think oral cancer is preventable?	249 (52)	230 (48)
	Aware about a Govt. programme for prevention of cancer	215 (45)	264 (55)
Attitude	Do you feel it gives you pleasure?	441 (92)	38 (8)
	Do you feel it relieves stress?	327 (68)	152 (32)
	Have you ever tried to quit?	86 (18)	393 (82)
	Will you quit if made aware of its harmful effects?	374 (78)	105 (22)
Practices	Are your family members using Tamul products?	397 (83)	82 (17)
	Anyone in the family asked not to chew Tamul?	209 (44)	270 (56)
	Are you thinking to quit?	74 (15)	405 (85)
	Have you ever tried to quit in last 6 months?	89 (19)	390 (81)
	Will you quit if came to know that Tamul causes cancer?	368 (77)	111 (23)
	Will you ask others to quit?	349 (73)	130 (27)
	Will you get screened for oral cancer?	394 (82)	85 (18)

**Table 4.** BQDS item frequencies, grouped by factor (*N* = 479).

Item	Current study		Herzog *et al* [11]	
	*N* (%)	Rank	%	Rank
**Factor 1: Physical and psychological urgent need**				
1. Cannot go on without areca nut	274 (57)	5	62	5
2. Difficulty in concentration after reducing dose	181 (38)	8	57	10
3. Experienced depression or drowsiness	127 (27)	11	52	11
4. The strong craving after reducing/stopping to chew	291 (61)	3	73	1
5. Spend time to find when not available	276 (58)	4	64	3
6. Travel great distance to find when not available	119 (25)	12	58	9
7. Felt agitated, irritated, or anxious after reducing	172 (36)	9	60	8
**Factor 2: Increasing dose**				
8. Trouble stopping once started chewing	295 (62)	1	67	2
9. Ever chewed non-stop	149 (31)	10	61	7
10. Gradually increased the amount of use after first use	292 (61)	2	64	4
11. Felt the need to increase the amount of use periodically	262 (55)	6	52	12
12. Often chewed betel nut/quid more than expected	193 (40)	7	62	6
**Factor 3: Maladaptive use**				
13. Continue chewing teeth loosen	78 (16)	14	39	14
14. Continue chewing if you had sensitive teeth	80 (17)	13	43	13
15. Continue chewing if experienced mouth ulcers	77 (16)	15	26	15
16. Reduced or given up activities because of chewing	75 (16)	16	17	16
